# Roles of Local Soluble Factors in Maintaining the Growth Plate: An Update

**DOI:** 10.3390/genes14030534

**Published:** 2023-02-21

**Authors:** Yiqian Zhang, Xenab Ahmadpoor, Hang Lin

**Affiliations:** 1 Department of Orthopaedic Surgery, University of Pittsburgh School of Medicine, 450 Technology Drive, Rm 217, Pittsburgh, PA 15219, USA; 2 Department of Bioengineering, University of Pittsburgh Swanson School of Engineering, 450 Technology Drive, Rm 217, Pittsburgh, PA 15219, USA

**Keywords:** growth plate, endochondral ossification, Wnt, β-catenin, micro physiological system, tissue chip, tissue engineering

## Abstract

The growth plate is a cartilaginous tissue found at the ends of growing long bones, which contributes to the lengthening of bones during development. This unique structure contains at least three distinctive layers, including resting, proliferative, and hypertrophic chondrocyte zones, maintained by a complex regulatory network. Due to its soft tissue nature, the growth plate is the most susceptible tissue of the growing skeleton to injury in childhood. Although most growth plate damage in fractures can heal, some damage can result in growth arrest or disorder, impairing leg length and resulting in deformity. In this review, we re-visit previously established knowledge about the regulatory network that maintains the growth plate and integrate current research displaying the most recent progress. Next, we highlight local secretary factors, such as Wnt, Indian hedgehog (Ihh), and parathyroid hormone-related peptide (PTHrP), and dissect their roles and interactions in maintaining cell function and phenotype in different zones. Lastly, we discuss future research topics that can further our understanding of this unique tissue. Given the unmet need to engineer the growth plate, we also discuss the potential of creating particular patterns of soluble factors and generating them in vitro.

## 1. Introduction

**Figure 1:**
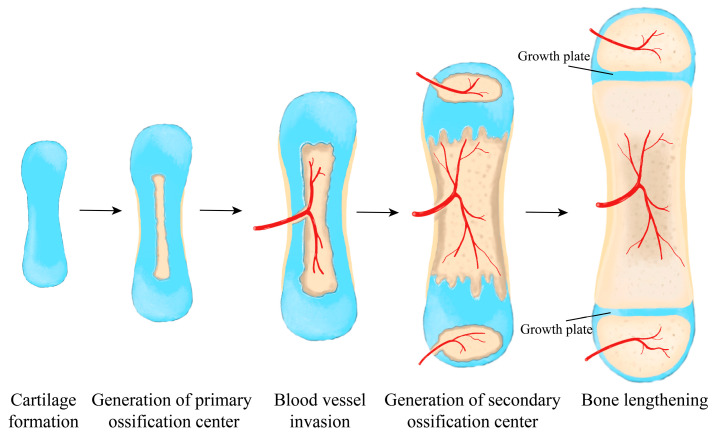
Major steps of the endochondral ossification. Invasion of blood vessels and bone cells leads to mineralization and generation of the primary (middle of the bone) and secondary (ends of the bone) ossification centers. The growth plates exist in between, contributing to bone lengthening.

During fetal development, endochondral ossification (Figure 1), a tightly regulated process of cell proliferation, chondrogenesis, hypertrophy, and ossification/apoptosis drives bone formation and growth. Blood vessels and osteogenic cells invade the formed cartilage template, which promotes further mineralization and generates the primary ossification center. As the fetus grows and the primary ossification center expands, a secondary ossification center forms at the ends of the developing bone. This results in an epiphyseal growth plate between the two ossification centers.

During development, the post-natal growth plate in humans controls bone lengthening until adulthood [1]. In contrast, a rodent’s growth plate does not disappear but loses ossification capacity when bones are mature [1]. The growth plate has a unique zonal structure. Based on cell morphology and phenotype, this soft tissue divides into three distinct zones that contain chondrocytes in resting, proliferative, or hypertrophic states. Closest to the epiphysis is the resting zone, primarily populated by parathyroid hormone-related protein (PTHrP)+ chondrocytes. Using a PTHrP-mCherry knock-in reporter allele, Mizuhashi et al. found that these chondrocytes originate from the perichondria region [2]. Recently, chondroprogenitor cells, such as skeletal stem cells, were also found in this area [2,3,4]. These multipotent cells can generate all types of chondrocytes of the growth plate. Furthermore, they maintain the proliferation of chondrocytes in a non-autonomous manner and delay their hypertrophic differentiation, thus maintaining the integrity of the growth plate.

Newton et al. further demonstrated that the chondroprogenitors in the resting zone are depleted during the fetal and neonatal periods but acquire the self-renewing capacity when the secondary ossification center is formed [4]. Surrounding tissues also regulate cells in the growth plate. For example, removing periosteal stem cells (PSCs) gradually impaired endochondral bone formation at the growth plate in post-natal mice. PSCs could maintain the proliferation of resting zone stem cells by producing Indian hedgehog (Ihh), compensating for the reduced Ihh production by growth plate chondrocytes during the progressive decline in growth with age and, consequently, ensure the post-natal bone growth [5]. In addition, the topmost of the resting zone, which is situated in proximity to the secondary ossification center, contains Forkhead box A2 (FoxA2) + stem cells. The FoxA2+ stem cells exhibit a greater ability to self-replicate than PTHrP+ stem cells and possess osteo-chondro-progenitor activity for growth plate regeneration [6]. These cells can also enter the resting zone and differentiate into columns of chondrocytes.

The proliferating zone is immediately adjacent to the resting zone. Chondrocytes in this area enter active cell replication resulting in pairs of cells in a single lacuna. With the generation of the cartilage matrix, the proliferated chondrocytes flatten, and cell clusters re-organize into columns parallel to the proximodistal axis of the bones. When proliferative chondrocytes reach terminal morphology and exit the cell cycle, they form the hypertrophic zone. These cells continue to enlarge, initiate ossification, and attract blood vessels and osteogenic cells to move in and remodel cartilage into bone. The hypertrophic chondrocytes can either evolve into osteoblasts and directly contribute to bone tissue formation or undergo apoptosis and leave the extracellular matrix as the template for bone formation [7]. Among the cells in the three layers, hypertrophic chondrocytes show high vulnerability to mechanical stress and are protected by a secondary ossification center from apoptosis caused by extensive loading [8].

At the periphery of the growth plate, immediately adjacent to the perichondrium, another chondrocyte population, called “borderline” chondrocytes, was recently identified [9]. These cells behave as transient mesenchymal precursor cells that can differentiate into osteoblasts and marrow stromal cells.

## 2. Overview of the Factors Maintaining the Growth Plate

**Figure 2:**
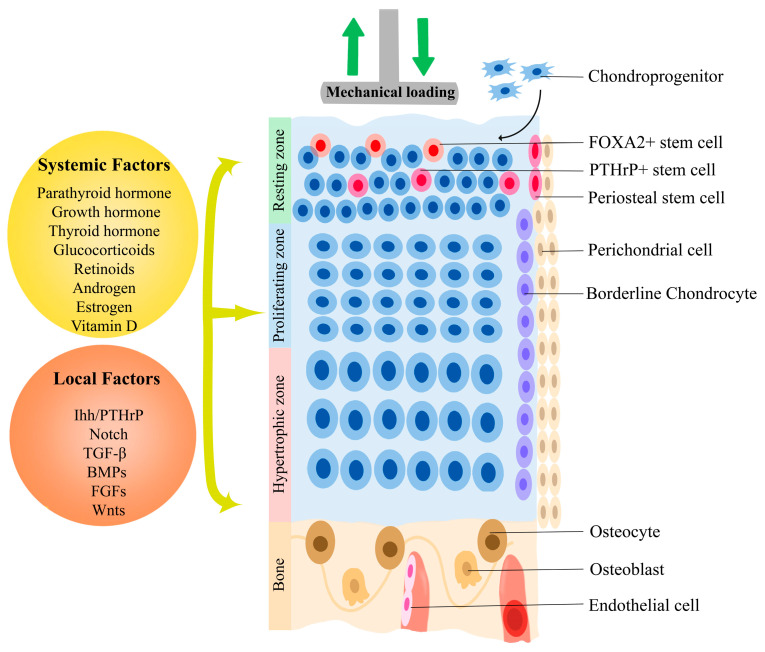
Local and systemic factors for maintaining the growth plate. Mechanical loading and systemic factors, including peptide hormones, steroid hormones, sex hormones, and local factors, form a delicate network to regulate the proliferation and hypertrophy of chondrocytes in the different zones. These local factors can be generated by chondrocytes within the growth plate, as well as by different types of surrounding cells.

The growth plate is controlled and regulated by surrounding environments (Figure 2), such as the periosteum [5], blood vessels [10], and mechanical stimulation [11], as well as internally generated biochemical factors by cells within the growth plate [12]. For example, stem cells in the perichondrium can regulate the stem cells within the resting zone through deriving Ihh to maintain endochondral bone formation, in addition to participating in intramembranous bone formation [5]. Moreover, endothelial cells of vessels release matrix metalloproteinase-9 (MMP9) to resorb the growth plate cartilage matrix [10]. In addition to these paracrine factors from surrounding tissues, mechanical loading affects the proliferation and hypertrophy of chondrocytes and columnar organization of the growth plate, with the reduction resulting in shorter limbs [13].

Mechanical stimuli activate Piezo1, a mechanically activated ion channel, and control the bone modeling phase [14]. Deleting Piezo1 has been shown to cause delayed skeletal maturity and remarkably reduced the secondary spongiosa but did not impair the morphology of the growth plate and primary ossification. Mechanical loading also stimulates the formation of a secondary ossification center, protecting the hypertrophic chondrocytes in the growth plate from potential injury due to high mechanical stress [8]. In addition to the protection from bone tissue, the growth-plate chondrocytes resist mechanical injuries through several mechanisms, such as inducing autophagy [15] and extension of the primary cilium [16,17]. For example, ciliary gene intraflagellar transport protein 88 (Ift88) knock-out (KO) mice showed narrow growth plate and endochondral ossification inhibition [16], demonstrating that Ift88 helped promote chondrocyte ciliation, cartilage resorption, mineralization, and, finally, coordinated ossification of growth plate from the disruptive mechanical force. Additionally, the Sprouty family, a modulator of the receptor tyrosine kinase signaling pathway, maintains the morphology of the primary cilium of chondrocytes in the growth plate [17]. The absence of Sprouty proteins displayed longer primary cilium and a shorter limb phenotype in mice.

This morphogenic process in the growth plate is also highly regulated by biochemical signaling pathways, which contain a wide variety of molecules systemically or locally generated, including parathyroid hormone [18], growth hormone (GH) [19], thyroid hormone [20], androgen [21], estrogen, vitamin D, glucocorticoids [22] and retinoids [23]. For example, lower levels of estrogen promote bone lengthening by inducing growth hormone secretion, while higher levels result in cellular senescence [24,25]. Vitamin D and its receptor maintain vessels and osteoblasts invasion in the hypertrophic zone during the bone formation process [26] and protect the growth plate from extreme strain [27]. Deleting glucocorticoid receptors was shown to delay cartilage maturation in mice, highlighting the effect endogenous glucocorticoids have on endochondral ossification [28]. Retinoids and retinoic acid receptors (RAR) are also important regulators of chondrocyte differentiation, with retinoids having a higher concentration in hypertrophic chondrocytes [23]. For instance, RAR agonists promoted hypertrophic-related gene expression [29], and, inversely, inhibiting RAR reduced hypertrophic differentiation of human mesenchymal stem cells (MSCs) [30]. The suppressor of cytokine signaling 2 (SOCS2), one of the members of the suppressor of cytokine signaling family glycoproteins, is an intracellular suppressor of the GH signaling pathway [31]. SOCS2-deficient mice that enhanced GH signaling pathway created wider proliferative zones of the growth plates [32].

**Figure 3:**
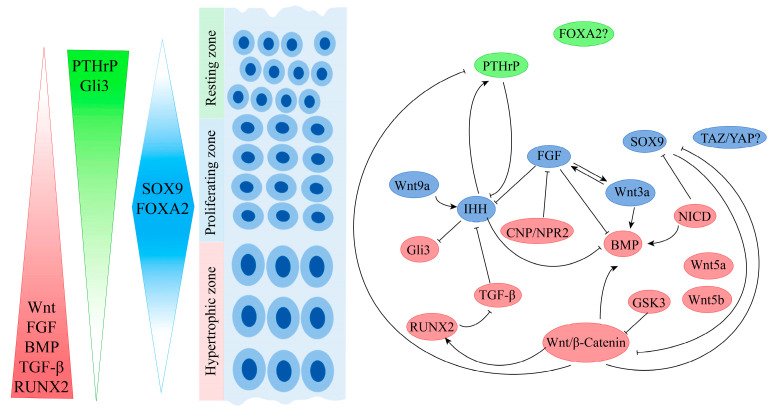
Distribution patterns and interactions of local factors in different zones of the growth plate. There are at least three types of molecular gradients. The molecules in red have the highest expression levels in the hypertrophic zone and the lowest in the resting zone. In contrast, the molecules in green have an opposite trend. The molecules in blue have the highest level in the proliferating zone and decrease in the resting and hypertrophic zones.

More information about the influences of biomechanical cues and systemically generated factors on the growth plate can be found in several recent review articles [12,33,34,35]. In addition to those systemic factors, several locally produced factors, including Ihh/PTHrP signaling, Notch signaling, BMPs, FGFs, TGF-B, and the Wnts, have previously been reported to play a pertinent role in the regulation of the formation and function of the growth plate, which is the focus of this review (Figure 3).

## 3. Local Factors for Maintaining the Growth Plate

The interaction of Ihh and PTHrP between the three zones is essential in regulating the cell phenotypes in different layers. PTHrP is primarily generated by resting zone cells as well as peripheral stem cells, functioning partially through inhibiting salt-inducible kinases and inducing histone deacetylase 4 (HDAC4) and HDAC5 [36] as a signal to keep chondrocytes in a proliferative state and inhibit differentiation to hypertrophy [33]. Ihh, synthesized by early hypertrophic chondrocytes and periosteal stem cells, maintains proliferation and inhibits hypertrophy of chondrocytes by promoting PTHrP expression [37,38]. Inhibiting Ihh signaling reduces chondrocyte proliferation and leads to premature hypertrophy, resulting in an impaired growth plate and limb shortening [39,40]. Previous research also shows that Ihh promotes the expression of PTHrP in the perichondrium and that Ihh^−/−^ mice lack expression of PTHrP. Thus, Ihh and PTHrP create a negative feedback loop and mutually control the proliferation and differentiation of growth plate chondrocytes.

Bone morphogenetic proteins (BMPs) and transforming growth factor-β (TGF-β) belong to the TGF-β superfamily, playing an essential role in cartilage formation and maintenance. The expression of BMP7 is mainly in the proliferative zone, while BMP2 and BMP6 are in the hypertrophic zone. BMP inhibitors accumulate in the resting zone to keep cells in a relatively static state [41]. These distinctive expressions of BMPs create a signaling gradient in the growth plate to induce chondrocyte maturation. Additionally, Ihh stimulates *BMP* expression, and BMP signaling regulates Ihh expression, thus creating a feedback loop [42]. The synergistic effects from Ihh and BMPs promote chondrocytes to leave the resting zone, where they are regulated by PTHrP signaling and undergo hypertrophic morphology.

TGF-β signaling can function through activating Smads or in a Smad-independent manner [43,44]. In the human growth plate, different TGF-β proteins exist in different zones. Specifically, TGF-β2 is expressed in all zones, with the lowest levels in the resting zone and the highest levels in the hypertrophic zones, while TGF-β1 is expressed only in the proliferating zones [45]. The function of TGF-β signaling is to promote chondrocyte proliferation rather than hypertrophic differentiation. Importantly, TGF-βs upregulate PTHrP and downregulate Ihh [45], implicating an interplay between TGF-β and Ihh/PTHrP signaling.

Fibroblast growth factors (FGFs) and corresponding receptors are distinctly expressed in three zones. The resting and hypertrophic zones contain low and high levels of FGF receptor-1 (FGFR1), respectively, while elevated levels of FGER3 exist in the proliferating zone [46]. Previous research shows that FGFR3 mutation in mice shortened the growth plate by suppressing chondrocyte proliferation and differentiation [47]. Similarly, Perlecan, a multifunctional extracellular proteoglycan, forms gradients within the tissue, like FGF-2 and FGF-18, to promote chondrocyte proliferation and differentiation into the column arrangements. Perlecan also has a mechanical conductive effect on chondrocytes by combining FGF-2. In contrast, FGF-18 promotes the osteogenesis of cells in the ossification center [43]. Minina et al. further demonstrated that FGF signaling has antagonistic effects on Ihh and BMP signaling by downregulating the expression of Ihh and BMPs [48].

The Notch signaling pathway is activated when ligands bind to Notch receptors and undergo several cleavages. The released Notch intracellular domain (NICD) interacts with the recombination signal binding protein-jk (RBPjk), a potent DNA-binding transcription factor, thus regulating the expression of downstream genes. Previous research shows that NICD accumulates in pre-hypertrophic and hypertrophic chondrocytes, and suppressing or overexpressing NICD results in the abnormal formation of the growth plate [49]. Furthermore, in the Notch gain of function of mice embryos, the activation of the Notch pathway notably decreased mRNA expression of SOX9, highlighting that Notch signaling suppresses the differentiation of hypertrophic chondrocytes by inhibiting SOX9 expression. Conversely, reducing the function of Notch signaling has higher SOX9 expression [49]. Therefore, rigorously controlled Notch signaling is needed to maintain proper bone development. In addition, reduced activation of Notch signaling suppresses BMP2 function in promoting hypertrophic differentiation, while activating Notch and BMP signaling induces chondrocyte hypertrophy and inhibits proliferation [50], suggesting that BMPs may be a downstream factor regulated by the Notch signaling.

Moreover, C-type natriuretic peptide (CNP) and its receptor NPR2 induce bone growth and interact with multiple signaling pathways in the growth plate [51]. Mutation of NPR2 causes thickening of the hypertrophic zone [52] and a series of skeletal deformities [53,54]. Recent research shows that the CNP/NPR2 pathway extends bone length by upgrading Guanosine 3′,5′-cyclic monophosphate (cGMP) levels, which activates Ca^2+^ channels and lets Ca^2+^ enter the chondrocytes and promotes proliferating zone extension [55]. In addition, CNP reduces FGF expression while promoting chondrocyte and osteoblast proliferation [56,57].

## 4. Wnt Signaling Pathway

Among the regulators in the growth plate, Wnt signaling serves a unique function. It simultaneously guides cell spatial features and position in a sequential column orientation [58]. Wnt signaling classifies as a canonical pathway that relies on the activation of β-catenin [59] or a non-canonical pathway, including Wnt/planar cell polarity (PCP) and Wnt/Ca^2+^. The canonical Wnt signaling pathway influences proliferation and differentiation in the growth plate through a combination of Wnts, the Wnt receptor complex Frizzled (FZD), and the lipoprotein receptor-related protein-5/6 (LRP5/6) [60]. Non-canonical Wnt/PCP signaling is also necessary for the growth plate. For example, disrupting the location of PCP proteins causes chondrocytes to lose polarity and delays the process of endochondral ossification [61].

Previous reports show that Wnt contains 19 members in humans, 9 of which exist in the growth plate, including Wnt 2b, 4, 5a, 5b, 7b, 9a, 10a, 10b, and 11 [62]. Hallett et al. recently reported that chondrocytes in the resting zone stay in a Wnt inhibitory environment to maintain static conditions. The activation of canonical Wnt signaling impaired the formation and expansion of chondrocytes in the resting zone and subsequently disrupted their differentiation into proliferating chondrocytes [63]. In contrast, expression levels of Wnts -4, -10b, -5a, -5b, and -11 significantly increase as chondrocytes differentiate into proliferative and pre-hypertrophic states [62]. The overexpression of Wnt4 in mice results in a larger hypertrophic zone and a smaller proliferating zone [64], indicating that Wnt4 contributes to the maturation of chondrocytes.

Research shows that Wnt3a can promote hypertrophic differentiation [65] of chondrocytes. In contrast, induction of Wnt16 suppressed the function of Wnt3a and resulted in less chondrocyte apoptosis [66]. In pre-hypertrophic chondrocytes, Wnt-9a regulates Ihh expression and promotes chondrocyte hypertrophy [67]. Wnt5a and Wnt5b function through non-canonical pathways, displaying a relative gradient in the three zones. Specifically, starting with low levels of Wnt5a and Wnt5b in the resting zone, their expression levels increase in the proliferative zone, promoting cell differentiation [62]. However, Wnt5a may have reversed roles in the hypertrophic zone as elevated levels of Wnt5a inhibits further chondrocyte maturation [68].

Recent reports show that Wnt/β-catenin regulates skeletal progenitors that reside in the outermost layer of the growth plate, contributing to the lateral growth of the growth plate in mice [69]. In addition, the Wnt/β-catenin responsive cells also exist in the resting and proliferating zone, which strongly indicates that Wnt/β-catenin is involved in growth plate expansion at the growing stage.

## 5. Interactions of Local Factors

All the previously mentioned factors dynamically interact to maintain the zonal structure of the growth plates and promote endochondral ossification. Thus, it is important to demonstrate their interactions [70,71,72]. In this article, given the critical hub function of the Wnt/β-catenin pathway in mediating several factors, we use it as the hub to demonstrate the interactions of di. The Wnt signaling pathway possesses multiple interactions with several pathways. First, the Wnt/β-catenin pathway has antagonistic effects with Ihh/PTHrP signaling in chondrocyte hypertrophy. Wnt/β-catenin signaling promotes initial hypertrophic differentiation of proliferating chondrocytes by inhibiting the PTHrP signaling rather than repressing PTHrP expression [73]. However, the Wnt/β-catenin pathway also regulates the terminal differentiation of the hypertrophic zone in a PTHrP-independent way. Wnt and Ihh signaling gene expression seem to be impacted by RUNX2 together [74], upregulating the downstream factor of RUNX2 to improve the proliferation of chondrocytes by activating the Wnt and Ihh signaling pathway simultaneously. A previous study showed that, when decreasing Wnt9a and β-catenin in embryos, the activation of Ihh signaling in early hypertrophic chondrocytes reduced, demonstrating that the canonical Wnt/β-catenin pathway can regulate Ihh expression through Wnt9a [67].

TGF-β collaborates with Wnt/β-catenin signaling by promoting the expression of Wnts and co-receptors [75,76]. Conversely, Wnt/β-catenin signaling can also promote the activity of TGF-β signaling by increasing the expression of TGFβRI, which also needs RUNX2 [77]. Increased Wnt3a promotes BMP2 and BMP4 expression in chondrocytes, indicating that Wnt/β-catenin signaling may promote BMP signaling and induce chondrocyte differentiation through the revitalization of BMP signaling [78]. Furthermore, Wnt/β-catenin signaling regulates BMP7. For instance, inhibiting Wnt/β-catenin signaling activity reduces BMP7 expression, resulting in decreased differentiation of mesenchymal stem cells into chondrocytes in rabbits [79].

There are synergistic effects between Wnt/β-catenin and FGF signaling pathways in discouraging chondrocyte differentiation [80]. Wnt3a and FGF2 work together to inhibit the formation of the chondrocyte extracellular matrix and regulate the cellular morphology of chondrocytes. Wnt/β-catenin signaling and SOX9 exit the mutually suppressive relationship during bone development. The Wnt/β-catenin signaling displays regulatory effects by inhibiting SOX9 expression by extrinsic Wnt ligands. Conversely, SOX9 inhibits Wnt/B-catenin through different mechanisms, one of which is to induce β-catenin phosphorylation and rapid degradation [81]. Glycogen synthase kinase (GSK) 3 signaling can negatively regulate the Wnt/β-catenin pathway, and deletion of GSK3 was shown to increase the activation of Wnt/β-catenin and lead to a series of adverse effects, including disorganization, apoptosis of chondrocytes, and a gene expression variation, and remolding of the growth plate [82].

## 6. Transcription Factors in the Growth Plate

The soluble factors influence cell phenotype and function through transcriptional factors and downstream genes. Several transcription factors control the formation and function of the growth plate (Figure 3). For example, Runt-related transcription factor 2 (RUNX2) promotes chondrocyte maturation and subsequent ossification [83]. The absence of RUNX2 inhibited the proper formation of the hypertrophic zone and impaired cartilage matrix degradation in some long bones of mice [84]. Specifically, RUNX2 has two isoforms, RUNX2-I and RUNX2-II. RUNX2-I, mostly expresses in the perichondrium and proliferative zones, plays a role in early osteoblastogenesis, while RUNX2-II mainly exists in hypertrophic zones and is necessary for terminal chondrocytes differentiation [85,86]. SOX9 plays a vital role in regulating chondrocytes in all zones to maintain proliferation and multiple sequence differentiation [87] and may play several roles in regulating TGF-β and BMP signaling. Deleting SOX9 inhibits TGF-β signaling and promotes BMP signaling. Gli3 acts as a downstream factor of Ihh, and reducing Gli3 by the overexpression of Ihh caused low levels of PTHrP and increased chondrocyte hypertrophy [37].

TAZ/YAP, a downstream factor of the Hippo pathway, controls the proliferation and maturation of chondrocytes by regulating the expression of SOX5 and Col10a1, respectively [88]. The loss of TAZ leads to the zones shortening the growth plate in mice. Hypoxia-inducible transcription factor (HIF)-1α is critical to the survival of chondrocytes. Previous studies have shown that deletion of HIF-1α in cartilage led to spatially unrestricted cell division and necrosis in the embryonic growth plate. Furthermore, recent studies have shown that inactivating HIF- led to a shorter growth plate and skeletal dysplasia, partially caused by an over-modification of collagen [89]. Recently, research states that heterogeneous nuclear ribonucleoprotein K (hnRNPK), an RNA-binding protein, can control HIF-1 by suppressing the overloading of glycolysis and premature differentiation of chondrocytes [90]. Other factors, FoxA2 and MEF2C, are both crucial regulators in promoting hypertrophic differentiation [91].

## 7. Summary and Future Perspectives

During development, the growth plate is an essential cartilaginous part in the endochondral ossification of the long bone. In this review, we summarize the latest understanding of the molecular network that maintains the structure of the growth plate. Of note, many of the factors talked about in this review were first defined years ago, while current studies primarily focus on revealing new functions and interactions with one another. We did not witness progression in identifying new regulatory factors. It is unclear whether currently known mechanisms are sufficient to maintain such a highly organized and dynamic tissue. The advances in elucidating a complete molecular regulatory network will rely on combining imaging, bio information, and genetic models. For example, Rubin et al. developed an imaging and analysis pipeline, called 3D Morphometric Analysis for Phenotypic significance (3D MAPs), which creates 3D morphology maps of the growth plate by using a procedure to clear tissue, segment chondrocytes, and project cells onto their locations [92]. Three-dimensional MAPs revealed continuous morphogenetic behaviors of chondrocytes within the growth plate. Another example includes using single-cell RNA sequencing, which has identified the relative expression of genes across different layers of cells [87]. Although this study focused on examining the role of SOX9 in growth factors, the same set of data can inform us of new factors not previously identified. One limitation of current single-cell sequencing is that it loses spatial information; however, it can be overcome by a spatially resolved transcriptomic method. These state-of-art technologies allow scientists to measure all the gene activity in a tissue sample and map where activity is occurring. To the best of our knowledge, relevant studies have not been conducted on the growth plate.

The goal of understanding the structure and biology of the growth plate is to treat disorders and injuries. The growth plate is susceptible to damage, such as bone fractures and congenital or acquired diseases [93]. Limb length inequality and deformities often happen in children with growth plate damage. Unfortunately, there are limited therapeutic methods [94]. Stem cell therapy can potentially repair the growth plate by supplementing progenitor cells, but current studies are still being tested on experimental animals [95]. Dai et al. recently used Antarctic Krill (AKPs) to improve long bone growth in mice by activating multiple pathways, such as GH, BMP, and Wnt signaling pathways [96]. However, further research is needed to fully understand the complexity of regulating and maintaining the growth plate to become an effective treatment.

Tissue engineering of the growth plate is another avenue to replace the damaged growth plate. For example, polyelectrolyte (PEC) complex hydrogels, which possess highly controlled mechanical properties, have been shown to affect the differentiation outcomes of MSCs to chondrogenic lineages in vitro and increase cartilage formation in vivo [97]. A 3D printed mimetic composite using a poly(ethylene glycol)-based resin showed to support the chondrogenesis of rabbit MSCs in vitro and enabled bone elongation, although it only presented limited growth plate formation [98]. Moreover, scaffold-combined stem cells could further regenerate cartilage. Aimed at creating a growth plate-like structure, Pitacco et al. loaded human MSCs into fibrin-based bio-inks and bio-printed them into polycaprolactone (PCL) frameworks. The constructs were capable of forming early hypertrophic constructs, supporting vascularization and mineralization, and, finally, contributing to endochondral bone regeneration [99]. Interestingly, extracellular matrix-mimic hydrogel loading exosomes derived from bone marrow mesenchymal stem cells could repair growth plate injury not only by regenerating chondrocytes and extracellular matrix but also by inhibiting local inflammatory response [100]. This study highlighted that, instead of becoming cells in the growth plate, MSCs can also be used to facilitate the generation of the growth plate. Tiffany et al. and Wang et al. recently reviewed relevant progress [93,101]. The representative in vitro and in vivo growth plate models are listed in Table 1.

**Figure 4:**
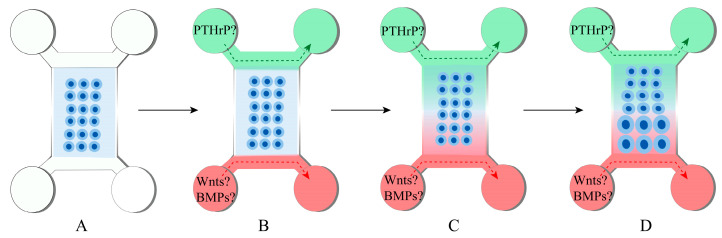
A proposed method to generate the growth plate in vitro. (**A**). The dual-flow bioreactor contains one central compartment and two channels. Stem cells, such as native or induced pluripotent stem cells (iPSC)-derived mesenchymal stem cells, will be encapsulated into a hydrogel scaffold within the central compartment. A chondrogenic medium will be perfused through the channels to induce chondrogenesis. (**B**) After cartilage is formed, a culture medium containing several factors will be introduced via two streams, indicated by green and red. (**C**) Through perfusion, gradients of these factors are expected to be established within the scaffold. (**D**) Cells at various locations will receive distinct stimulations and acquire different phenotypes, generating a zonal structure within the bioreactor.

Although making scientific strides, current methods have not been able to make a complete growth plate. Given the critical role of the abovementioned pattern of distinct factors, from one end to the other, in maintaining the growth plate, a bioreactor with dual flow capacity can be a powerful tool when engineering this tissue. In Figure 4, we propose a developmentally informed method to generate a growth plate in vitro. Initially, the progenitors are loaded uniformly into a 3D-printed scaffold, where two mediums with different components are introduced, inducing cells to differentiate into chondrocytes. Due to the variety of signals received, these cells will mimic the varying phenotype of chondrocytes in the resting, proliferative, and hypertrophic zones of the native growth plate. If necessary, using elastic scaffolds and platforms will allow the extension of the growth plate.

## Figures and Tables

**Table 1:** Recent progress in engineering the growth plate.

Model	Cell Type	Growth Factor	Scaffold	Results	Reference
Rat and in vitro	Rat BMSCs		3D GMOCS hydrogel	Promoted the chondrogenic differentiation in vitro Promoted cartilage regeneration in vivo	[102]
Rat	Human BMSCs	TGF-β3	3D bio-printed into PCL frameworks	Formed early hypertrophic constructs and supported vascularization and mineralization,	[99]
Rat and in vitro	Human MSCs (unknown type)	TGF-β1	Alginate-chitosan PEC hydrogels	Formed chondrogenic lineages in vitro increase cartilage formation in vivo	[97]
Rabbit and in vitro	Rabbit BMSCs	TGF-β3	3D printed mimetic composite	Supported chondrogenesis in vitro enabled bone elongation in vivo	[98]
Rat	Rat BMSCs		GMOCS hydrogel	Regenerated chondrocytes and extracellular matrix, inhibited local inflammatory response	[100]
Rat			Alginate-chitosan hydrogels	Produced the most cartilage-like tissue in 50:50 of irradiated alginate and chitosan	[103]
Rabbit	Rabbit BMSCs or chondrocytes		Cell sheets	Resumed bone growth	[104]
In vitro	Mouse chondrocytes	PTH1–34, IHH	Alginate Hydrogel	Established a tunable, 3D growth plate model	[105]
Rabbit	Rabbit BMSCs		ECM	regenerated neogenetic chondrocytes	[106]
Rabbit		IGF-1	PLGA	Regenerated cartilage	[107]
Rabbit	BMCs	IGF-1	PLGA	Increased chondrocyte population	[108]
Rabbit	BMSCs		Chitosan	Promoted MSCs concentration	[109]

GMOCS: hydrogel formed from methacrylate-based gelatin (GM) and aldehyde-functionalized chondroitin sulfate (OCS) by dynamic Schiff base bonding under UV light. Exos: exosomes. ECM: extracellular matrix. PLGA: poly(lactic-co-glycolic acid) BMCs: bone marrow cells.

## Data Availability

Not applicable.

## References

[1] Lamuedra A., Gratal P., Calatrava L., Ruiz-Perez V.L., Largo R., Herrero-Beaumont G. (2020). Disorganization of chondrocyte columns in the growth plate does not aggravate experimental osteoarthritis in mice. Sci. Rep..

[2] Mizuhashi K., Ono W., Matsushita Y., Sakagami N., Takahashi A., Saunders T.L., Nagasawa T., Kronenberg H.M., Ono N. (2018). Resting zone of the growth plate houses a unique class of skeletal stem cells. Nature.

[3] Lui J.C. (2020). Home for a rest: Stem cell niche of the postnatal growth plate. J. Endocrinol..

[4] Newton P.T., Li L., Zhou B., Schweingruber C., Hovorakova M., Xie M., Sun X., Sandhow L., Artemov A.V., Ivashkin E. (2019). A radical switch in clonality reveals a stem cell niche in the epiphyseal growth plate. Nature.

[5] Tsukasaki M., Komatsu N., Negishi-Koga T., Huynh N.C.-N., Muro R., Ando Y., Seki Y., Terashima A., Pluemsakunthai W., Nitta T. (2022). Periosteal stem cells control growth plate stem cells during postnatal skeletal growth. Nat. Commun..

[6] Muruganandan S., Pierce R., Teguh D.A., Perez R.F., Bell N., Nguyen B., Hohl K., Snyder B.D., Grinstaff M.W., Alberico H. (2022). A FoxA2+ long-term stem cell population is necessary for growth plate cartilage regeneration after injury. Nat. Commun..

[7] Yang L., Tsang K.Y., Tang H.C., Chan D., Cheah K.S.E. (2014). Hypertrophic chondrocytes can become osteoblasts and osteocytes in endochondral bone formation. Proc. Natl. Acad. Sci. USA.

[8] Xie M., Gol’Din P., Herdina A.N., Estefa J., Medvedeva E.V., Li L., Newton P.T., Kotova S., Shavkuta B., Saxena A. (2020). Secondary ossification center induces and protects growth plate structure. Elife.

[9] Mizuhashi K., Nagata M., Matsushita Y., Ono W., Ono N. (2019). Growth Plate Borderline Chondrocytes Behave as Transient Mesenchymal Precursor Cells. Bone Miner. Res..

[10] Romeo S.G., Alawi K.M., Rodrigues J., Singh A., Kusumbe A.P., Ramasamy S.K. (2019). Endothelial proteolytic activity and interaction with non-resorbing osteoclasts mediate bone elongation. Nat. Cell Biol..

[11] Ueki M., Tanaka N., Tanimoto K., Nishio C., Honda K., Lin Y.-Y., Tanne Y., Ohkuma S., Kamiya T., Tanaka E. (2008). The effect of mechanical loading on the metabolism of growth plate chondrocytes. Ann. Biomed. Eng..

[12] Agirdil Y. (2020). The growth plate: A physiologic overview. EFORT Open Rev..

[13] Killion C.H., Mitchell E.H., Duke C., Serra R. (2017). Mechanical loading regulates organization of the actin cytoskeleton and column formation in postnatal growth plate. Mol. Biol. Cell.

[14] Hendrickx G., Fischer V., Liedert A., von Kroge S., Haffner-Luntzer M., Brylka L., Pawlus E., Schweizer M., Yorgan T., Baranowsky A. (2021). Piezo1 Inactivation in Chondrocytes Impairs Trabecular Bone Formation. J. Bone Miner. Res..

[15] Zhang J.-M., Wang Z.-G., He Z.-Y., Qin L., Wang J., Zhu W.-T., Qi J. (2022). Cyclic mechanical strain with high-tensile triggers autophagy in growth plate chondrocytes. J. Orthop. Surg. Res..

[16] Coveney C.R., Samvelyan H.J., Miotla-Zarebska J., Carnegie J., Chang E., Corrin C.J., Coveney T., Stott B., Parisi I., Duarte C. (2022). Ciliary IFT88 Protects Coordinated Adolescent Growth Plate Ossification From Disruptive Physiological Mechanical Forces. J. Bone Miner. Res..

[17] Hruba E., Kavkova M., Dalecka L., Macholan M., Zikmund T., Varecha M., Bosakova M., Kaiser J., Krejci P., Hovorakova M. (2021). Loss of Sprouty Produces a Ciliopathic Skeletal Phenotype in Mice Through Upregulation of Hedgehog Signaling. J Bone Miner. Res..

[18] Hirai T., Chagin A.S., Kobayashi T., Mackem S., Kronenberg H.M. (2011). Parathyroid hormone/parathyroid hormone-related protein receptor signaling is required for maintenance of the growth plate in postnatal life. Proc. Natl. Acad. Sci. USA.

[19] De Luca F. (2020). Regulatory role of NF-κB in growth plate chondrogenesis and its functional interaction with Growth Hormone. Mol. Cell Endocrinol..

[20] Gouveia C.H., Miranda-Rodrigues M., Martins G.M., Neofiti-Papi B. (2018). Thyroid Hormone and Skeletal Development. Vitam. Horm..

[21] Vanderschueren D., Vandenput L., Boonen S., Lindberg M.K., Bouillon R., Ohlsson C. (2004). Androgens and bone. Endocr. Rev..

[22] Lui J.C., Baron J. (2011). Effects of glucocorticoids on the growth plate. Endocr. Dev..

[23] Pacifici M. (2018). Retinoid roles and action in skeletal development and growth provide the rationale for an ongoing heterotopic ossification prevention trial. Bone.

[24] Allen D.B., Merchant N., Miller B.S., Backeljauw P.F. (2021). Evolution and Future of Growth Plate Therapeutics. Horm. Res. Paediatr..

[25] Kubo N., Awada T., Hirose N., Yanoshita M., Takano M., Nishiyama S., Tsuboi E., Kita D., Ito S., Nakatani A. (2022). Longitudinal effects of estrogen on mandibular growth and changes in cartilage during the growth period in rats. Dev. Biol..

[26] Verlinden L., Carmeliet G. (2021). Integrated View on the Role of Vitamin D Actions on Bone and Growth Plate Homeostasis. JBMR Plus.

[27] Wang J., Kokinos B.P., Lang P.J., Crenshaw T.D., Henak C.R. (2022). Vitamin D deficiency and anatomical region alters porcine growth plate properties. J. Biomech..

[28] Rapp A.E., Hachemi Y., Kemmler J., Koenen M., Tuckermann J., Ignatius A. (2018). Induced global deletion of glucocorticoid receptor impairs fracture healing. Faseb. J..

[29] Shimo T., Koyama E., Okui T., Masui M., Kunisada Y., Ibaragi S., Yoshioka N., Kurio N., Yoshida S., Sasaki A. (2019). Retinoic Receptor Signaling Regulates Hypertrophic Chondrocyte-specific Gene Expression. In Vivo.

[30] Riedl M., Witzmann C., Koch M., Lang S., Kerschbaum M., Baumann F., Krutsch W., Docheva D., Alt V., Pfeifer C. (2020). Attenuation of Hypertrophy in Human MSCs via Treatment with a Retinoic Acid Receptor Inverse Agonist. Int. J. Mol. Sci..

[31] Pass C., MacRae V.E., Huesa C., Ahmed S.F., Farquharson C. (2012). SOCS2 is the critical regulator of GH action in murine growth plate chondrogenesis. J. Bone Miner. Res..

[32] Samvelyan H.J., Huesa C., Cui L., Farquharson C., Staines K.A. (2022). The role of accelerated growth plate fusion in the absence of SOCS2 on osteoarthritis vulnerability. Bone Jt. Res..

[33] Kronenberg H.M. (2003). Developmental regulation of the growth plate. Nature.

[34] Hallett S.A., Ono W., Ono N. (2019). Growth Plate Chondrocytes: Skeletal Development, Growth and Beyond. Int. J. Mol. Sci..

[35] Samsa W.E., Zhou X., Zhou G. (2017). Signaling pathways regulating cartilage growth plate formation and activity. Semin. Cell Dev. Biol..

[36] Nishimori S., Wein M.N., Kronenberg H.M. (2021). PTHrP targets salt-inducible kinases, HDAC4 and HDAC5, to repress chondrocyte hypertrophy in the growth plate. Bone.

[37] Ohba S. (2020). Hedgehog Signaling in Skeletal Development: Roles of Indian Hedgehog and the Mode of Its Action. Int. J. Mol. Sci..

[38] St-Jacques B., Hammerschmidt M., McMahon A.P. (1999). Indian hedgehog signaling regulates proliferation and differentiation of chondrocytes and is essential for bone formation. Genes Dev..

[39] Xiu C., Gong T., Luo N., Ma L., Zhang L., Chen J. (2022). Suppressor of fused-restrained Hedgehog signaling in chondrocytes is critical for epiphyseal growth plate maintenance and limb elongation in juvenile mice. Front. Cell Dev. Biol..

[40] Cong L., Jiang P., Wang H., Huang L., Wu G., Che X., Wang C., Li P., Duan Q., Guo X. (2022). MiR-1 is a critical regulator of chondrocyte proliferation and hypertrophy by inhibiting Indian hedgehog pathway during postnatal endochondral ossification in miR-1 overexpression transgenic mice. Bone.

[41] Nilsson O., Parker E.A., Hegde A., Chau M., Barnes K.M., Baron J. (2007). Gradients in bone morphogenetic protein-related gene expression across the growth plate. J. Endocrinol..

[42] Minina E., Wenzel H.M., Kreschel C., Karp S., Gaffield W., McMahon A.P., Vortkamp A. (2001). BMP and Ihh/PTHrP signaling interact to coordinate chondrocyte proliferation and differentiation. Development.

[43] Tzavlaki K., Moustakas A. (2020). TGF-β Signaling. Biomolecules.

[44] Wu M., Chen G., Li Y.-P. (2016). TGF-β and BMP signaling in osteoblast, skeletal development, and bone formation, homeostasis and disease. Bone Res..

[45] Li T.F., O’Keefe R.J., Chen D. (2005). TGF-β signaling in chondrocytes. Front. Biosci..

[46] Ornitz D.M., Marie P.J. (2015). Fibroblast growth factor signaling in skeletal development and disease. Genes Dev..

[47] Naski M., Colvin J., Coffin J., Ornitz D. (1998). Repression of hedgehog signaling and BMP4 expression in growth plate cartilage by fibroblast growth factor receptor 3. Development.

[48] Minina E., Kreschel C., Naski M.C., Ornitz D., Vortkamp A. (2002). Interaction of FGF, Ihh/Pthlh, and BMP signaling integrates chondrocyte proliferation and hypertrophic differentiation. Dev. Cell..

[49] Mead T.J., Yutzey K.E. (2009). Notch pathway regulation of chondrocyte differentiation and proliferation during appendicular and axial skeleton development. Proc. Natl. Acad. Sci. USA.

[50] Shang X., Wang J., Luo Z., Wang Y., Morandi M.M., Marymont J.V., Hilton M.J., Dong Y. (2016). Notch signaling indirectly promotes chondrocyte hypertrophy via regulation of BMP signaling and cell cycle arrest. Sci. Rep..

[51] Rintz E., Węgrzyn G., Fujii T., Tomatsu S. (2022). Molecular Mechanism of Induction of Bone Growth by the C-Type Natriuretic Peptide. Int. J. Mol. Sci..

[52] Yamamoto K., Kawai M., Yamazaki M., Tachikawa K., Kubota T., Ozono K., Michigami T. (2019). CREB activation in hypertrophic chondrocytes is involved in the skeletal overgrowth in epiphyseal chondrodysplasia Miura type caused by activating mutations of natriuretic peptide receptor B. Hum. Mol. Genet..

[53] Irfanullah, Zeb A., Shinwari N., Shah K., Gilani S.Z.T., Khan S., Lee K.W., Raza S.I., Hussain S., Liaqat K. (2018). Molecular and in silico analyses validates pathogenicity of homozygous mutations in the NPR2 gene underlying variable phenotypes of Acromesomelic dysplasia, type Maroteaux. Int. J. Biochem. Cell Biol..

[54] Amano N., Kitoh H., Narumi S., Nishimura G., Hasegawa T. (2020). A novel NPR2 mutation (p.Arg388Gln) in a patient with acromesomelic dysplasia, type Maroteaux. Clin. Pediatr. Endocrinol..

[55] Miyazaki Y., Ichimura A., Kitayama R., Okamoto N., Yasue T., Liu F., Kawabe T., Nagatomo H., Ueda Y., Yamauchi I. (2022). C-type natriuretic peptide facilitates autonomic Ca(2+) entry in growth plate chondrocytes for stimulating bone growth. eLife.

[56] Krejci P., Masri B., Fontaine V., Mekikian P.B., Weis M., Prats H., Wilcox W.R. (2005). Interaction of fibroblast growth factor and C-natriuretic peptide signaling in regulation of chondrocyte proliferation and extracellular matrix homeostasis. J. Cell Sci..

[57] Chen W.X., Liu H.H., Li R.X., Mammadov G., Wang J.J., Liu F.F., Samadli S., Wu Y.F., Zhang D.D., Luo H.H. (2020). C-type natriuretic peptide stimulates osteoblastic proliferation and collagen-X expression but suppresses fibroblast growth factor-23 expression in vitro. Pediatr. Rheumatol. Online J..

[58] Loh K.M., van Amerongen R., Nusse R. (2016). Generating Cellular Diversity and Spatial Form: Wnt Signaling and the Evolution of Multicellular Animals. Dev. Cell..

[59] Yang Y. (2012). Wnt signaling in development and disease. Cell Biosci..

[60] Nusse R., Clevers H. (2017). Wnt/β-Catenin Signaling, Disease, and Emerging Therapeutic Modalities. Cell.

[61] Wan Y., Szabo-Rogers H.L. (2021). Chondrocyte Polarity During Endochondral Ossification Requires Protein-Protein Interactions Between Prickle1 and Dishevelled2/3. J. Bone Miner. Res..

[62] Andrade A.C., Nilsson O., Barnes K.M., Baron J. (2007). Wnt gene expression in the post-natal growth plate: Regulation with chondrocyte differentiation. Bone.

[63] Hallett S.A., Matsushita Y., Ono W., Sakagami N., Mizuhashi K., Tokavanich N., Nagata M., Zhou A., Hirai T., Kronenberg H.M. (2021). Chondrocytes in the resting zone of the growth plate are maintained in a Wnt-inhibitory environment. eLife.

[64] Lee H.H., Behringer R.R. (2007). Conditional expression of Wnt4 during chondrogenesis leads to dwarfism in mice. PLoS ONE.

[65] Bertrand J., Kräft T., Gronau T., Sherwood J., Rutsch F., Lioté F., Dell’Accio F., Lohmann C.H., Bollmann M., Held A. (2020). BCP crystals promote chondrocyte hypertrophic differentiation in OA cartilage by sequestering Wnt3a. Ann. Rheum. Dis..

[66] Yan H., Hu Y., Akk A., Rai M.F., Pan H., Wickline S.A., Pham C.T. (2020). Induction of WNT16 via Peptide-mRNA Nanoparticle-Based Delivery Maintains Cartilage Homeostasis. Pharmaceutics.

[67] Später D., Hill T.P., O’Sullivan R.J., Gruber M., Conner D.A., Hartmann C. (2006). Wnt9a signaling is required for joint integrity and regulation of Ihh during chondrogenesis. Development.

[68] Hosseini-Farahabadi S., Geetha-Loganathan P., Fu K., Nimmagadda S., Yang H.J., Richman J.M. (2013). Dual functions for WNT5A during cartilage development and in disease. Matrix Biol..

[69] Usami Y., Gunawardena A.T., Francois N.B., Otsuru S., Takano H., Hirose K., Matsuoka M., Suzuki A., Huang J., Qin L. (2019). Possible Contribution of Wnt-Responsive Chondroprogenitors to the Postnatal Murine Growth Plate. J. Bone Miner. Res..

[70] Long F., Ornitz D.M. (2013). Development of the endochondral skeleton. Cold Spring Harb. Perspect. Biol..

[71] Michigami T. (2013). Regulatory mechanisms for the development of growth plate cartilage. Cell Mol. Life Sci..

[72] Chen H., Tan X.N., Hu S., Liu R.Q., Peng L.H., Li Y.M., Wu P. (2021). Molecular Mechanisms of Chondrocyte Proliferation and Differentiation. Front. Cell Dev. Biol..

[73] Guo X., Mak K.K., Taketo M.M., Yang Y. (2009). The Wnt/β-catenin pathway interacts differentially with PTHrP signaling to control chondrocyte hypertrophy and final maturation. PLoS ONE.

[74] Matsuura V.K.S.K., Yoshida C.A., Komori H., Sakane C., Yamana K., Jiang Q., Komori T. (2020). Expression of a Constitutively Active Form of Hck in Chondrocytes Activates Wnt and Hedgehog Signaling Pathways, and Induces Chondrocyte Proliferation in Mice. Int. J. Mol. Sci..

[75] Zhou S., Eid K., Glowacki J. (2004). Cooperation between TGF-β and Wnt pathways during chondrocyte and adipocyte differentiation of human marrow stromal cells. J. Bone Miner. Res..

[76] Li T.-F., Chen D., Wu Q., Chen M., Sheu T.-J., Schwarz E.M., Drissi H., Zuscik M., O’Keefe R.J. (2006). Transforming growth factor-β stimulates cyclin D1 expression through activation of β-catenin signaling in chondrocytes. J. Biol. Chem..

[77] McCarthy T.L., Centrella M. (2010). Novel links among Wnt and TGF-β signaling and Runx2. Mol. Endocrinol..

[78] Chen M., Zhu M., Awad H., Li T.-F., Sheu T.-J., Boyce B.F., Chen D., O’Keefe R.J. (2008). Inhibition of β-catenin signaling causes defects in postnatal cartilage development. J. Cell Sci..

[79] Fu H.D., Wang H.R., Li D.H. (2017). BMP-7 accelerates the differentiation of rabbit mesenchymal stem cells into cartilage through the Wnt/β-catenin pathway. Exp. Ther. Med..

[80] Buchtova M., Oralova V., Aklian A., Masek J., Vesela I., Ouyang Z., Obadalova T., Konecna Z., Spoustova T., Pospisilova T. (2015). Fibroblast growth factor and canonical WNT/β-catenin signaling cooperate in suppression of chondrocyte differentiation in experimental models of FGFR signaling in cartilage. Biochim. Biophys. Acta.

[81] Topol L., Chen W., Song H., Day T., Yang Y. (2009). Sox9 inhibits Wnt signaling by promoting β-catenin phosphorylation in the nucleus. J. Biol. Chem..

[82] Bali S.K., Bryce D., Prein C., Woodgett J.R., Beier F. (2021). Glycogen synthase kinase 3 α/β deletion induces precocious growth plate remodeling in mice. J. Mol. Med..

[83] Komori T. (2022). Whole Aspect of Runx2 Functions in Skeletal Development. Int. J. Mol. Sci..

[84] Rashid H., Chen H., Javed A. (2021). Runx2 is required for hypertrophic chondrocyte mediated degradation of cartilage matrix during endochondral ossification. Matrix Biol. Plus.

[85] Xiao Z.S., Hjelmeland A.B., Quarles L.D. (2004). Selective deficiency of the “bone-related” Runx2-II unexpectedly preserves osteoblast-mediated skeletogenesis. J. Biol. Chem..

[86] Zhang S., Xiao Z., Luo J., He N., Mahlios J., Quarles L.D. (2009). Dose-dependent effects of Runx2 on bone development. J. Bone Miner. Res..

[87] Haseeb A., Kc R., Angelozzi M., de Charleroy C., Rux D., Tower R.J., Yao L., da Silva R.P., Pacifici M., Qin L. (2021). SOX9 keeps growth plates and articular cartilage healthy by inhibiting chondrocyte dedifferentiation/osteoblastic redifferentiation. Proc. Natl. Acad. Sci. USA.

[88] Li Y., Yang S., Qin L., Yang S. (2021). TAZ is required for chondrogenesis and skeletal development. Cell Discov..

[89] Stegen S., Laperre K., Eelen G., Rinaldi G., Fraisl P., Torrekens S., Van Looveren R., Loopmans S., Bultynck G., Vinckier S. (2019). HIF-1α metabolically controls collagen synthesis and modification in chondrocytes. Nature.

[90] Chen Y., Wu J., Zhang S., Gao W., Liao Z., Zhou T., Li Y., Su D., Liu H., Yang X. (2022). Hnrnpk maintains chondrocytes survival and function during growth plate development via regulating Hif1α-glycolysis axis. Cell Death Dis..

[91] Tan Z., Niu B., Tsang K.Y., Melhado I.G., Ohba S., He X., Huang Y., Wang C., McMahon A.P., Jauch R. (2018). Synergistic co-regulation and competition by a SOX9-GLI-FOXA phasic transcriptional network coordinate chondrocyte differentiation transitions. PLoS Genet..

[92] Rubin S., Agrawal A., Stegmaier J., Krief S., Felsenthal N., Svorai J., Addadi Y., Villoutreix P., Stern T., Zelzer E. (2021). Application of 3D MAPs pipeline identifies the morphological sequence chondrocytes undergo and the regulatory role of GDF5 in this process. Nat. Commun..

[93] Tiffany A.S., Harley B.A.C. (2022). Growing Pains: The Need for Engineered Platforms to Study Growth Plate Biology. Adv. Healthc. Mater..

[94] Sananta P., Lesmana A., Alwy Sugiarto M. (2022). Growth plate injury in children: Review of literature on PubMed. J. Public Health Res..

[95] Sananta P., Isnansyah Y., Rosandi R.D., Sugiarto M.A. (2022). The Management Growth Plate Injury in Animal Studies with Stem Cells Technique: Systematic Review. Acta Inform Med..

[96] Dai Y., Li Z., Fu M., Li Y., Xue C., Wang J. (2021). Peptides from Euphausia superba Promote Longitudinal Bone Growth by Accelerating Growth Plate Chondrocyte Proliferation and Hypertrophy. Curr. Pharm. Biotechnol..

[97] Stager M.A., Thomas S.M., Rotello-Kuri N., Payne K.A., Krebs M.D. (2022). Polyelectrolyte Complex Hydrogels with Controlled Mechanics Affect Mesenchymal Stem Cell Differentiation Relevant to Growth Plate Injuries. Macromol. Biosci..

[98] Yu Y., Fischenich K.M., Schoonraad S.A., Weatherford S., Uzcategui A.C., Eckstein K., Muralidharan A., Crespo-Cuevas V., Rodriguez-Fontan F., Killgore J.P. (2022). A 3D printed mimetic composite for the treatment of growth plate injuries in a rabbit model. NPJ Regen. Med..

[99] Pitacco P., Sadowska J.M., O’Brien F.J., Kelly D.J. (2023). 3D bioprinting of cartilaginous templates for large bone defect healing. Acta Biomater..

[100] Guan P., Liu C., Xie D., Mao S., Ji Y., Lin Y., Chen Z., Wang Q., Fan L., Sun Y. (2022). Exosome-loaded extracellular matrix-mimic hydrogel with anti-inflammatory property Facilitates/promotes growth plate injury repair. Bioact. Mater..

[101] Wang X., Li Z., Wang C., Bai H., Wang Z., Liu Y., Bao Y., Ren M., Liu H., Wang J. (2021). Enlightenment of Growth Plate Regeneration Based on Cartilage Repair Theory: A Review. Front. Bioeng. Biotechnol..

[102] Guan P., Ji Y., Kang X., Liu W., Yang Q., Liu S., Lin Y., Zhang Z., Li J., Zhang Y. (2023). Biodegradable Dual-Cross-Linked Hydrogels with Stem Cell Differentiation Regulatory Properties Promote Growth Plate Injury Repair via Controllable Three-Dimensional Mechanics and a Cartilage-like Extracellular Matrix. ACS Appl. Mater. Interfaces.

[103] Erickson C.B., Newsom J.P., Fletcher N.A., Yu Y., Rodriguez-Fontan F., Weatherford S.A., Hadley-Miller N., Krebs M.D., Payne K.A. (2021). Anti-VEGF antibody delivered locally reduces bony bar formation following physeal injury in rats. J. Orthop. Res..

[104] Gültekin A., Ağirdil Y., Duman B., DEMİR C.S., Karaöz E. (2020). Comparison of mesenchymal stem cell sheets and chondrocyte sheets in a rabbit growth plate injury model. Turk. J. Med. Sci..

[105] Erickson A.G., Laughlin T.D., Romereim S.M., Sargus-Patino C.N., Pannier A.K., Dudley A.T. (2018). A Tunable, Three-Dimensional In Vitro Culture Model of Growth Plate Cartilage Using Alginate Hydrogel Scaffolds. Tissue Eng. Part A.

[106] Li W., Xu R., Huang J., Bao X., Zhao B. (2017). Treatment of rabbit growth plate injuries with oriented ECM scaffold and autologous BMSCs. Sci. Rep..

[107] Sundararaj S.K.C., Cieply R.D., Gupta G., Milbrandt T.A., Puleo D.A. (2015). Treatment of growth plate injury using IGF-I-loaded PLGA scaffolds. J. Tissue Eng. Regen. Med..

[108] Clark A., Hilt J.Z., Milbrandt T.A., Puleo D.A. (2015). Treating Proximal Tibial Growth Plate Injuries Using Poly(Lactic-co-Glycolic Acid) Scaffolds. Biores. Open Access.

[109] Azarpira M.R., Shahcheraghi G.H., Ayatollahi M., Geramizadeh B. (2015). Tissue engineering strategy using mesenchymal stem cell-based chitosan scafolds in growth plate surgery: A preliminary study in rabbits. Orthop. Traumatol. Surg. Res..

